# Appropriate definition of childbirth using Japanese administrative database: a cross-sectional cohort validation study

**DOI:** 10.1186/s12884-026-08797-9

**Published:** 2026-02-11

**Authors:** Miyuki Koizumi, Hiroki Nakajima, Yuichi Nishioka, Emiri Morita, Tomoya Myojin, Tatsuya Noda, Tomoaki Imamura, Yutaka Takahashi

**Affiliations:** 1 Department of Diabetes and Endocrinology, Nara Medical University, 840 Shijo- cho, Kashihara, Nara 634-8522 Japan; 2 Department of Public Health, Health Management and Policy, Nara Medical University, 840 Shijo-cho, Kashihara, Nara, 634-8521 Japan; 3 Department of Community Health and Preventive Medicine, Hamamatsu University School of Medicine, Shizuoka, Japan; 4 Department of Hygiene and Public Health, Kansai Medical University, Osaka, Japan

**Keywords:** Claims data, Childbirth, Validation

## Abstract

**Background:**

Claims data analyses are useful in clinical research. However, evidence on the validity of claims-based algorithms for identifying childbirth remains limited, particularly in settings where mother–child linkage is unavailable. Therefore, we aimed to develop and validate algorithms to identify childbirth from a claims database.

**Methods:**

The DeSC database, including claims data for approximately 13 million people, was accessed. Eighteen algorithms were designed using combinations of diagnosis-related codes with/without a suspected flag regarding childbirth ([A+susp]/[A]), medical procedure codes [B], and medication codes [C]. We used the parent–child identifier (ID) in the DeSC database as the gold standard, which is assigned based on family relationship information recorded in the insurer-managed registry of insured persons. Parent–child ID links children to an insured parent within the same insurance unit, enabling mother–child linkage. The gold standard for the month and year of childbirth was defined as the child’s month and year of birth among women aged 15–49 years linked by parent–child IDs during the observation period. We calculated sensitivity, specificity, positive predictive value (PPV), negative predictive value (NPV), Kappa Index, and Youden Index for each algorithm. To validate algorithms for estimating second childbirth during the observation period, which would become useful in defining childbirth, identification of second childbirth began 2–24 months after the first, given that the average age difference was two years.

**Results:**

A total of 854,626 women were included in this study, of whom 37,934 were aged 15–49 years at the time of parent–child ID assignment and classified as experiencing childbirth during the observation period. The algorithm with the highest value was “[A+susp] or [B] or [C]” (sensitivity: 66.9%, specificity: 98.9%, PPV: 73.7%, NPV: 98.5%, Kappa Index: 0.69, and Youden Index: 0.66). With respect to second childbirth, algorithm “[A+susp] or [B] or [C]” showed that the 11-month difference had the highest Youden Index at 0.57.

**Conclusion:**

We developed algorithms based on claims data and established an optimal algorithm for estimating childbirth. This validated algorithm can be used for accurate estimation of childbirth to clarify pregnancy- and childbirth-related diseases in future claims database studies.

**Supplementary Information:**

The online version contains supplementary material available at 10.1186/s12884-026-08797-9.

## Background

Administrative claims databases provide longitudinal data, including medical inpatient claims, medical outpatient claims, medication use, and major procedures [1]. The use of administrative claims data in epidemiological research has increased in recent years, reflecting the value of these real-world data sources [2]. Claims data are not collected for study purposes but, rather, for processing medical reimbursements; however, claims data can be useful in bridging gaps in clinical information regarding diagnoses, examination results, and disease severity [3]. Therefore, verifying the validity of the information recorded in claims data is essential [4]. Claims-based definitions have been developed and validated for various outcomes, including diabetes-related complications (e.g., coronary artery disease), and cause of death [5, 6]. However, validation studies regarding the definition of diseases and conditions remain limited.

Claims data related to pregnancy and the postpartum period have high potential as useful real-world data. Studies conducted using claims data related to pregnancy, childbirth, and the postpartum period have revealed various issues such as infant maltreatment, pertussis vaccination rates among parents with infants, prenatal care, postpartum depression, pregnancy- and lactation-associated osteoporosis, and use of antiepileptic drugs in pregnancy [7–12]. In these studies, the date of childbirth was identified using claims data for medical procedure or diagnosis-related codes regarding childbirth, hospitalisations for childbirth, and in some studies, linkage to birth certificate record [13–18]. The ConcePTION project uses a composite set of European data sources from multiple institutions, including claims databases, and evaluated pregnancy start and end dates [19]. The validity of these algorithms has been evaluated against external reference sources, such as medical record review and/or linked birth certificate data, as well as other approaches including expert review or comparisons with official statistics. However, these datasets were validated either only among women who gave birth or by confirming agreement between descriptive statistics and other data sources; consequently, they were unable to present sensitivity and specificity within claims databases. For example, a Japanese validation study that attempted to identify dates of childbirth using a claims database examined claims data only among obstetrics patients from a single institution; selection bias was acknowledged, and sensitivity and specificity could not be estimated.

Therefore, we aimed to develop and validate algorithms that identify the month and year of childbirth from claims data among women with family-relationship information, regardless of childbirth history, using a large, population-representative claims database. A claims database containing insurer-derived family information was used, and algorithm validity was assessed by comparing claims-based estimates of the month and year of childbirth with the linkage-based gold standard derived from the family information.

## Methods

### Study design

A cross-sectional observational analysis was performed using the DeSC database (DeSC Healthcare, Tokyo, Japan). The database comprised data of women whose husbands were identified from family information between 2014 and 2023. This study was approved by the Ethics Committee of Nara Medical University (approval no. 1123) and conducted in accordance with the principles outlined in the Declaration of Helsinki. Only anonymized secondary administrative claims data provided for research purposes were used; therefore, the requirement for informed consent to participate was waived by the Ethics Committee of Nara Medical University.

### Data sources

The DeSC database is a nationwide administrative claims database that captures a subset of insured individuals in Japan, comprising data from the Advanced Elderly Medical Service System, health insurance associations established by companies (with approval of the Japanese government) for private-sector employees and public servants, and National Health Insurance for self-employed people and freelancers. This database was constructed using monthly claims from medical institutions and pharmacies, specific health checkups, and registries in Japan submitted between April 2014 and January 2023, including approximately 13 million insured people (10.5% of the Japanese population). The population representativeness of the DeSC database has been evaluated previously and reported to be broadly comparable to national population estimates [20]. The DeSC database provides information on encrypted personal identifiers (IDs), month and year of birth, sex, International Classification of Diseases, Tenth Revision (ICD-10) codes, medication codes (name, dose, and administration period), and medical procedure codes for insured people. In Japanese administrative claims, diagnosis codes can be accompanied by a “suspected flag,” indicating that the diagnosis is recorded as a provisional/rule-out condition (e.g., to support reimbursement for diagnostic tests and related care) and is typically distinguished from definitive diagnoses. In Japan, claims data may not be a comprehensive source of data concerning pregnancy and childbirth, given that uncomplicated natural births (i.e., uncomplicated normal vaginal deliveries/spontaneous vaginal births without obstetric interventions, with neither medical procedure codes nor medication codes regarding childbirth) are often not covered by medical insurance and would not show a recorded claim. Therefore, identifying childbirth using claims data has the limitation that it cannot capture uncomplicated vaginal births that generate no reimbursed claims. The deliveries without childbirth-related procedure codes and/or maternal delivery diagnosis codes may not be completely captured by claims-based identification.

Parent–child IDs are available in the DeSC database; however, they can be extracted only for insurers that have consented to the use of parent–child IDs. Parent–child IDs are assigned based on family-relationship information recorded in the registration of the insured person and maintained by insurers for eligibility management purposes. Because this registry information is operational data directly linked to benefits administration and eligibility management, a generally high level of data integrity can be expected. In the DeSC database, we can distinguish whether family information including parent–child IDs are available for each individual. When family information is available, the presence of a parent–child ID indicates that the individual has at least one child enrolled under the same insurance, whereas its absence indicates that the individual has no children enrolled under the same insurance. Parent–child IDs are not assigned to adopted children. Parent–child IDs are assigned only for live births; therefore, induced abortions, miscarriages, and stillbirths cannot be identified. In this study, linking the child’s month and year of birth to the mother using parent–child IDs enabled identification of the month and year of childbirth as an event in the mother’s medical record.

### Childbirth algorithm

In previous claims-based studies, childbirth has been identified using diagnosis and/or procedure codes and/or medicine codes [9, 11, 18, 21, 22]. In this study, algorithms were constructed using three coding domains: diagnosis-related codes, medical procedure codes, and medication codes regarding childbirth. In terms of the names of condition or disease that occurred just before delivery or during delivery and childbirth, the codes that appeared during delivery and childbirth were selected. The specific diagnosis-related codes are listed in Supplementary Tables 1, and codes corresponding to O14–15, O32–34, O42, O45, O48, O60–69, O70–72, O74–75, O80–84, O86–87, O90, O99, and Z37–8 in the International Classification of Diseases, Tenth Revision (ICD-10) were selected. Procedure codes were used to identify delivery-related interventions (e.g., labour induction, vacuum extraction, forceps delivery, and caesarean section; Supplementary Table 2). In addition, medication codes for oxytocin and dinoprostone—used for labour induction/augmentation and postpartum uterine contraction—were extracted (Supplementary Table 2). Our childbirth algorithm targeted deliveries that did not include codes for induced abortion or miscarriage. In claims data, distinguishing live births from stillbirths based solely on claims codes was difficult. Code lists and algorithm components were curated by obstetricians at Nara Medical University and combined to construct the final algorithms.

Eighteen algorithms for the claims-based definition of childbirth were designed. Algorithms 1–18 were constructed using a combination of three elements: [A] childbirth-related diagnosis-related codes recorded without a suspected flag or [A+susp] the same diagnosis-related codes recorded with a suspected flag; [B] childbirth-related medical procedure codes; and [C] childbirth-related medication codes (Table 1). In Japan, the rate of preterm births in 2022 was approximately 5.6% [23]. Additionally, previous reports have shown that the concordance rate between the algorithm and the gold standard is relatively low for preterm births [13]. Data of women with preterm births might influence analysis results. We created diagnosis-related codes regarding childbirth excluding potentially preterm births by excluding codes such as O45, O60, and O69, which represent placental abruption, preterm birth, and umbilical cord complications (Supplementary Table 3). We defined [A(pre(-))] as these diagnosis-related codes without a suspected flag and [A(pre(-))+susp] as those with a suspected flag. As described above, we combined conditions [B] and [C] to construct Algorithms 19–32 (Supplementary Table 4).

**Table 1:** Algorithms for identifying childbirth

Condition [A+susp]	Diagnosis-related codes regarding childbirth with a suspected flag
Condition [A]	Diagnosis-related codes regarding childbirth without a suspected flag
Condition [B]	Medical procedure codes regarding childbirth
Condition [C]	Medication codes regarding childbirth
Algorithm	Condition
Algorithm1	**[A+susp]**
Algorithm2	**[B]**
Algorithm3	**[C]**
Algorithm4	**[A+susp]** and **[B]**
Algorithm5	**[A+susp]** or **[B]**
Algorithm6	**[A+susp]** and **[C]**
Algorithm7	**[A+susp]** or **[C]**
ALgoritum8	**[B]** and **[C]**
Algorithm9	**[B]** or** [C]**
Algorithm10	**[A+susp] **and** [B] **and **[C]**
Algorithm11	**[A+susp]** or **[B] **or** [C]**
Algorithm12	**[A]**
Algorithm13	**[A]** and **[B]**
Algorithm14	**[A]** or **[B]**
Algorithm15	**[A]** and **[C]**
Algorithm16	**[A] **or** [C]**
Algorithm17	**[A]** and** [B] **and** [C]**
Algorithm18	**[A]** or **[B]** or** [C]**

Bold text indicates the algorithm conditions. *Susp *Suspected flag

The latest month and year recorded in the claims data for diagnosis-related codes, medical procedure codes or medication codes regarding childbirth were defined as the month and year of childbirth. In the algorithms used in this study, women with a single childbirth contributed that event, whereas women with multiple childbirths contributed only the most recent childbirth. The month of discharge may follow the month of childbirth, and the month of childbirth and that of diagnosis-related codes, medical procedure codes or medication codes may differ by 1 month. Therefore, “the month and year of childbirth from algorithms or 1 month before the month and year of childbirth from algorithms” and “the month and year of childbirth from the parent–child ID” were considered equivalent.

### The gold standard for month and year of childbirth

The gold standard for month and year of childbirth was defined as the month and year of birth of a child whose birth mother was aged 15–49 years and the mother and child were linked by parent–child IDs during the observation period. The provision of this information regarding family relationships was strictly reviewed from the perspective of personal data protection. The use of data from the DeSC database was permitted because the gold standard information was indispensable for the execution of this validation study. Because the parent–child ID does not reflect stillbirths, these were not included in this definition. In Japan, 99.9% of live births in 2022 occurred among women aged 15–49 years [23]. Therefore, age in this study was limited to 15–49 years to accurately determine the date of childbirth from the parent–child ID.

In our analysis, we included only women for whom the husband could be identified. This restriction was applied because, when spouses are enrolled in different insurance plans, a child may be registered as a dependent of the father and linked only to the father, and a parent–child ID may not be generated for the mother. In such cases, women may have childbirth-related diagnosis and/or procedure codes, but the linkage-based reference (gold standard) childbirth month and year cannot be ascertained; including these cases could therefore lead to erroneous classification of algorithm-identified deliveries as false positives, which would lead to an underestimation of algorithm performance.

### Statistical analysis

#### Identifying childbirth

The sensitivity, specificity, positive predictive value (PPV), negative predictive value (NPV), Kappa Index, and Youden Index for each algorithm with corresponding 95% confidence intervals were computed. Sensitivity and specificity were calculated by identifying whether the month and year of giving birth (-1 ~ 0 month) defined by the algorithms coincided with that of childbirth from the parent–child ID. PPV was the proportion of women identified by an algorithm as experiencing childbirth who had truly given birth. NPV was the proportion of women identified by an algorithm as not experiencing childbirth, who had not truly given birth. The Kappa Index was calculated for the agreement between each algorithm and the gold standard to identify the algorithms that maximised Kappa [24]. The Youden Index was calculated as (sensitivity + specificity) – 1, to weigh sensitivity and specificity equally.

### Identifying second childbirth during the observation period

Diagnosis-related codes regarding childbirth may be used repeatedly for prepartum and postpartum visits. Diagnosis-related and medical procedure codes for the same childbirth may appear in different months. In identifying multiple childbirths in the database, there is a risk of misrepresenting single childbirths as multiple childbirths within a short period. The number of months that passed after the consecutive diagnosis-related codes, medical procedure codes or medication codes may indicate another childbirth, rather than the same one. To confirm whether women with parent–child IDs had more than two childbirths using algorithms during the observation period, the validity of the difference in months based on each algorithm was verified: each month from 2 to 24 months after the first childbirth, given that the average age difference was reported to be two years in Japan in 2022 [23]. The Youden Index, sensitivity, specificity, PPV, NPV, and Kappa Index for each algorithm in each month for second childbirth were calculated. All analyses were performed using the Microsoft SQL Server 2022 Standard ^®^ (Microsoft Corp., Redmond, WA, USA) and IBM SPSS for Windows (version 29.0; IBM Corp., Armonk, NY, USA).

## Results

### Participant characteristics

In total, 854,626 women with husbands were identified in the DeSC database based on family information from the parent–child IDs. Table 2 presents the characteristics of the women included in this study. At the time of parent–child ID assignment, 37,934 women aged 15 to 49 (validation cohort birth rate, 4.4%) were classified as experiencing childbirth during the observation period (Supplementary Table 5). Sixty-eight registered women with a parent-child ID were ineligible, as they did not fall within the target registrant age range of 15–49 years (0.2% of women with parent–child IDs).

**Table 2:** Characteristics of women in the validation study

Age	Number	Percentage
0～4	7,162	0.8%
5～9	7,201	0.8%
10～14	7,505	0.9%
15～19	10,278	1.2%
20～24	20,312	2.4%
25～29	30,870	3.6%
30～34	44,518	5.2%
35～39	53,633	6.3%
40～44	62,345	7.3%
45～49	68,259	8.0%
50～54	65,660	7.7%
55～59	80,542	9.4%
60～64	150,979	17.7%
65～69	193,158	22.6%
70～74	52,204	6.1%
Total	854,626	100%

### Validating algorithms [1–18] for childbirth

Table 3 presents the accuracy of the administrative data algorithms in identifying women with childbirth experience and the month and year of their deliveries based on the DeSC database. The accuracy assessment of Algorithm 11, using diagnosis-related codes with a suspected flag or medical procedure codes or medication codes regarding childbirth, showed the highest value, with a sensitivity of 66.9%, specificity of 98.9%, PPV of 73.7%, NPV of 98.5%, Kappa Index of 0.69, and Youden Index of 0.66. Algorithm 18, which was created using diagnosis-related codes without a suspected flag or medical procedure codes or medication codes, resulted in a sensitivity of 65.0%, specificity of 98.9%, PPV of 74.0%, NPV of 98.4%, Kappa Index of 0.68, and Youden Index of 0.64.

**Table 3:** Accuracy of administrative data algorithms for identifying women with childbirth experience and the month and year of their deliveries

Algorithm	TP	TN	FN	FP	Sensitivity(%)	95% CI		Specificity (%)	95% CI		PPV (%)	95% CI		NPV (%)	95% CI		Kappa	Youden
Algorithm1	21857	809174	16077	7518	57.6%	57.1%	58.1%	99.1%	99.1%	99.1%	74.4%	73.9%	74.9%	98.1%	98.0%	98.1%	0.64	0.57
Algorithm2	15123	811960	22811	4732	39.9%	39.4%	40.4%	99.4%	99.4%	99.4%	76.2%	75.6%	76.8%	97.3%	97.2%	97.3%	0.51	0.39
Algorithm3	18275	810683	19659	6009	48.2%	47.7%	48.7%	99.3%	99.2%	99.3%	75.3%	74.7%	75.8%	97.6%	97.6%	97.7%	0.57	0.47
Algorithm4	11805	813103	26129	3589	31.1%	30.7%	31.6%	99.6%	99.5%	99.6%	76.7%	76.0%	77.4%	96.9%	96.8%	96.9%	0.43	0.31
Algorithm5	24960	808123	12974	8569	65.8%	65.3%	66.3%	99.0%	98.9%	99.0%	74.4%	74.0%	74.9%	98.4%	98.4%	98.4%	0.69	0.65
Algorithm6	14949	811989	22985	4703	39.4%	38.9%	39.9%	99.4%	99.4%	99.4%	76.1%	75.5%	76.7%	97.2%	97.2%	97.3%	0.50	0.39
Algorithm7	24991	807959	12943	8733	65.9%	65.4%	66.4%	98.9%	98.9%	99.0%	74.1%	73.6%	74.6%	98.4%	98.4%	98.5%	0.68	0.65
Algorithm8	13083	812797	24851	3895	34.5%	34.0%	35.0%	99.5%	99.5%	99.5%	77.1%	76.4%	77.7%	97.0%	97.0%	97.1%	0.46	0.34
Algorithm9	20230	809885	17704	6807	53.3%	52.8%	53.8%	99.2%	99.1%	99.2%	74.8%	74.3%	75.3%	97.9%	97.8%	97.9%	0.61	0.52
Algorithm10	10157	813629	27777	3063	26.8%	26.3%	27.2%	99.6%	99.6%	99.6%	76.8%	76.1%	77.5%	96.7%	96.7%	96.7%	0.38	0.26
Algorithm11	25361	807660	12573	9032	66.9%	66.4%	67.3%	98.9%	98.9%	98.9%	73.7%	73.3%	74.2%	98.5%	98.4%	98.5%	0.69	0.66
Algorithm12	20950	809610	16984	7082	55.2%	54.7%	55.7%	99.1%	99.1%	99.2%	74.7%	74.2%	75.2%	97.9%	97.9%	98.0%	0.62	0.54
Algorithm13	11680	813147	26254	3545	30.8%	30.3%	31.3%	99.6%	99.6%	99.6%	76.7%	76.0%	77.4%	96.9%	96.8%	96.9%	0.42	0.30
Algorithm14	24226	808497	13708	8195	63.9%	63.4%	64.3%	99.0%	99.0%	99.0%	74.7%	74.3%	75.2%	98.3%	98.3%	98.4%	0.68	0.63
Algorithm15	14819	812028	23115	4664	39.1%	38.6%	39.6%	99.4%	99.4%	99.4%	76.1%	75.5%	76.7%	97.2%	97.2%	97.3%	0.50	0.38
Algorithm16	24259	808335	13675	8357	64.0%	63.5%	64.4%	99.0%	99.0%	99.0%	74.4%	73.9%	74.9%	98.3%	98.3%	98.4%	0.67	0.63
Algorithm17	10052	813659	27882	3033	26.5%	26.1%	26.9%	99.6%	99.6%	99.6%	76.8%	76.1%	77.5%	96.7%	96.6%	96.7%	0.38	0.26
Algorithm18	24662	808019	13272	8673	65.0%	64.5%	65.5%	98.9%	98.9%	99.0%	74.0%	73.5%	74.5%	98.4%	98.4%	98.4%	0.68	0.64

*TP *True Positive, *TN *True Negative, *FP *False Positive, *FN *False Negative, *95% CI* 95% confidence interval, *PPV *positive predictive value, *NPV *negative predictive value

### Validating algorithms [19–32] for identifying childbirth, excluding potentially preterm birth

Supplementary Table 6 shows the accuracy of the administrative data algorithms in identifying women with childbirth experience and the month and year of their deliveries excluding those with a high possibility of preterm birth. Algorithm 25, which was created using diagnosis-related codes with a suspected flag or medical procedure code or medication codes, showed the highest value, with a sensitivity of 66.4%, specificity of 98.9%, PPV of 74.1%, NPV of 98.4%, Kappa Index of 0.69, and Youden Index of 0.65. Algorithm 32, which was created using diagnosis-related codes without a suspected flag or medical procedure codes or medication codes, resulted in a sensitivity of 64.9%, specificity of 99.0%, PPV of 74.2%, NPV of 98.4%, Kappa Index of 0.68, and Youden Index of 0.64.

### Validating algorithms for identifying second childbirth

Supplementary Table 7 shows the indicators of accuracy of the administrative data algorithms in identifying second childbirth when the evaluation of the algorithms began 2–24 months after the first childbirth during the observation period. Algorithm 11, when observation of diagnosis-related codes started 11 months after the first childbirth, showed a sensitivity of 59.8%, specificity of 97.4%, PPV of 80.6%, NPV of 93.0%, Kappa Index of 0.64, and the highest Youden Index of 0.57.

## Discussion

In this study, we established and validated algorithms for defining the month and year of childbirth based on a claims database among women, regardless of their childbirth history. Algorithm 11 involving diagnosis-related codes with a suspected flag or medical procedure codes or medication codes regarding childbirth yielded the highest value, with a Kappa Index of 0.69 and a sensitivity of 66.9% for all childbirths. The PPV was 73.7% in all algorithms, with the highest value being 77.1%, showing minimal difference. Algorithm 25 excluded births with a high possibility of being preterm from Algorithm 11. Algorithms 11 and 25 showed a nearly similar Youden Index at 0.66 and 0.65, respectively. Considering second childbirth using the claims database, at the start of observation, 11 months after the first childbirth, Algorithm 11 yielded the highest Youden Index at 0.57. Although sensitivity was not high at 59.8%, specificity, PPV, and NPV were high at 97.4%, 80.6%, and 93.0%, respectively.

Algorithms 1–18 yielded PPV and NPV of approximately 75% and 97%, respectively, for all childbirths. Algorithm 11, with the highest Kappa Index at 0.69, was the most accurate for identifying childbirth based on claims data. Comparing algorithms 11 and 18, using diagnosis-related codes with a suspected flag yielded a higher sensitivity than without a suspected flag, and both categories had an almost similar specificity. The PPV values of Algorithms 11 and 18 were nearly identical, which is due to the fact that there were very few cases of diagnosis-related codes with a suspected flag regarding childbirth among non-pregnant women.

In addition, we established and validated an algorithm for estimating the date of second childbirth. In the analysis, 11 months after the first childbirth, Algorithm 11 yielded the highest Youden Index at 0.57. Women who do not breastfeed after childbirth can ovulate and become pregnant 6 weeks after childbirth [10]. Considering that a baby is born at full term in 37–41 weeks, it takes approximately 11 months to give birth again and at least 6 weeks to become pregnant again, which may explain why the 11-month Youden Index was the highest.

In previous studies, the identification of the date of childbirth has varied depending on the data sources used, such as childbirth-related claims data, hospitalisations for childbirth, and birth certificate records, and algorithms for defining childbirth have typically been validated against electronic medical records or birth certificate records [7–10, 13–16]. The ConcePTION project developed an open-source pregnancy episode identification algorithm that reconciles pregnancy-related records across multiple electronic healthcare data sources (e.g., registries, primary care records, and hospital administrative records) mapped to a common data model to identify pregnancies, thereby enabling multi-country analyses [19]. In contrast, we evaluated childbirth identification in the DeSC claims database using routine claims data alone and conducted the validation in a broad population of women aged 15–49 years, including those with and without delivery-related claims, rather than restricting the analysis to women with an identified pregnancy or delivery episode. In Japan, a prior validation study assessed an algorithm using medical claims data from obstetric patients at a single institution [17, 18]. Such institution-based validations necessarily focus on women who gave birth and may not fully represent the broader population captured in routine claims databases. In that study, the PPV for live births was 98.1%, which is higher than 77.1% for Algorithm 8, the algorithm with the highest PPV in our study. In the previous study, the analysis was restricted to women receiving obstetric care at a tertiary medical center, and live births were distinguished from stillbirths and miscarriages. In contrast, our study included a nationwide population that also comprised women without childbirth, and misclassification of diagnosis-related codes and the frequent assignment of childbirth-related claims diagnoses at postnatal outpatient visits may have contributed to lower PPV. In addition, for procedure codes and medication codes, stillbirths or miscarriages may not generate a parent–child ID, yet procedures and medications may still be provided, potentially leading to false-positive classifications.

The proportions of mothers in different age ranges at the time of childbirth were as follows: 15–19 years (1.0%), 20–24 (8.6%), 25–29 (24.1%), 30–34 (34.7%), 35–39 (24.7%), 40–44 (6.6%), and 45–49 (0.3%). Proportions of age groups in our study correspond well with the proportions recorded nationally in Japan in 2022: 15–19 years (0.6%), 20–24 (6.9%), 25–29 (26.3%), 30–34 (36.3%), 35–39 (23.8%), 40–44 (6.0%), and 45–49 (0.2%). We can conclude from this similarity that the study cohort reflects the general population in Japan.

An important strength of this study is that it provides one of the largest linkage-based validations of claims-based childbirth identification, as it includes a total of 854,626 women regardless of childbirth history during the observation period. Using mother–child linkage via parent–child IDs as the reference, we evaluated a practical claims-only algorithm to estimate the month and year of childbirth. This validation framework is directly relevant to settings in which parent–child linkage is not available. Because the algorithm is based on routinely recorded diagnosis, procedure and medication codes in claims data, it should be applicable to other claims databases that lack parent–child IDs.

Notwithstanding the high accuracy of the framework we have developed in this study, there are several limitations that should be mentioned. First, the validation analysis only included women with the parent–child IDs as family information. Therefore, abortions, miscarriages, and stillbirths were outside the scope of this study, and algorithm performance for these outcomes could not be assessed. In addition, restricting the cohort to women with identifiable husbands (i.e., within the same family ID) may limit generalizability to families in which mother–child linkage cannot be established on the maternal side due to different insurance enrollment. However, the proportion of women who gave birth was almost the same as the national average; therefore, bias was considered small. Second, women with normal deliveries could not be evaluated by the algorithms in this study because medical insurance is generally not used for normal deliveries. The algorithms used in this study cannot identify childbirths without medical procedures. Therefore, when targeting normal deliveries that do not involve medical procedure codes or diagnosis-related codes regarding childbirth, it is necessary to use administrative databases such as parent–child IDs. Third, there may have been a misallocation of parent–child IDs when linking parent–child relationships in the administrative database. However, the possibility of a link error was considered extremely low because four women aged under 15 (0.02%) and 64 women aged over 50 (0.01%) years were identified as mothers by parent–child IDs.

## Conclusions

In this study, we established an optimal algorithm for estimating childbirth based on claims data. This validated algorithm will be useful for accurate estimation of childbirth in future claims database studies.

## Supplementary Information

Supplementary Material 1.

Supplementary Material 2.

Supplementary Material 3.

Supplementary Material 4.

Supplementary Material 5.

Supplementary Material 6.

Supplementary Material 7.

## Data Availability

The data that support the findings of this study are available from DeSC Healthcare, Inc., but restrictions apply to the availability of these data, which were used under the licence for the current study and are not publicly available. However, the data are available from the authors upon reasonable request and with permission from DeSC Healthcare, Inc.

## Abbreviations

ICD-10: International classification of diseases, tenth revision
ID: Identifier
TP: True positive
TN: True negative
FP: False positive
FN: False negative
PPV: Positive predictive value
NPV: Negative predictive value
95% CI: 95% Confidence interval
susp: Suspected flag
pre: Preterm birth
